# Estimation of Spatial-Temporal Gait Parameters Using a Low-Cost Ultrasonic Motion Analysis System

**DOI:** 10.3390/s140815434

**Published:** 2014-08-20

**Authors:** Yongbin Qi, Cheong Boon Soh, Erry Gunawan, Kay-Soon Low, Rijil Thomas

**Affiliations:** School of Electrical and Electronic Engineering, Nanyang Technological University, 50 Nanyang Avenue, Singapore 639798, Singapore; E-Mails: qiyo0001@e.ntu.edu.sg (Y.Q.); egunawan@ntu.edu.sg (E.G.); ekslow@ntu.edu.sg (K.-S.L.); rijil001@e.ntu.edu.sg (R.T.)

**Keywords:** ultrasonic sensor, gait analysis, walking assessment, gait kinematics, wireless sensor network

## Abstract

In this paper, a low-cost motion analysis system using a wireless ultrasonic sensor network is proposed and investigated. A methodology has been developed to extract spatial-temporal gait parameters including stride length, stride duration, stride velocity, stride cadence, and stride symmetry from 3D foot displacements estimated by the combination of spherical positioning technique and unscented Kalman filter. The performance of this system is validated against a camera-based system in the laboratory with 10 healthy volunteers. Numerical results show the feasibility of the proposed system with average error of 2.7% for all the estimated gait parameters. The influence of walking speed on the measurement accuracy of proposed system is also evaluated. Statistical analysis demonstrates its capability of being used as a gait assessment tool for some medical applications.

## 1. Introduction

The significance of spatial-temporal gait parameters measurement has been addressed in many research papers [1–3]. The quantitative analysis of such gait parameters can be helpful to diagnose impairments in balance control [4], monitor the progress in rehabilitation [5], and predict the risk of falling [6,7]. Such parameters include stride length, walking velocity, stride cadence, stride duration and asymmetry of stride. In particular, stride asymmetry has been shown to be more indicative of the underlying impairments and walking stability [8,9]. Therefore, having instruments that are capable of measuring these gait parameters about the patients' walking ability is useful in many clinical applications [10].

The most commonly employed method for gait analysis involves the use of multi-camera motion capture system and force plates, which is capable of measuring ground reaction forces and tracking the 3-dimensional positions of reflective markers [11]. However, measurements using this system require specialized laboratories, complex calibration and expensive equipments [12], which makes it ill-suited for routine applications. Moreover, it is sensitive to changes in lighting, clutter and shadow [13,14].

Many motion analysis systems using non-traditional methods have been proposed over the last decade [11]. These systems, for example, use wearable force sensors to measure the ground reaction force for the estimation of foot dynamics and centre of mass displacement [1,15,16]. Even X-ray is used to measure the 3-dimensional body segment parameters for gait analysis [17]. Since in many applications it is desirable to monitor human body motion in various environments, some portable and low-cost systems are preferred.

Inertial/magnetic systems are becoming more popular due to their low cost, small form factor and easy implementation [12]. However, when it comes to the estimates of foot displacements, double integration of measured accelerations is needed to get the displacement or position information. Unfortunately, it is difficult to obtain accurate motion accelerations because of the presence of sensor bias and measurement noise, which leads to the exponential increase of displacement error over integration time [18]. This issue can be mitigated either by applying some techniques to correct it periodically, such as zero velocity update (ZUPT), or by applying Kalman filter [19], or by combining with other sensors, such as imaging sensors, Radio Frequency identification (RFID) technology, or ultra-wide band (UWB) technique [20–23]. These mentioned hybrid motion tracking systems can improve the tracking accuracy, but with an increased cost, complexity of experiment installation and maintenance.

Ultrasonic sensors are among the most commonly used techniques in gait analysis due to its safety, low cost, and high accuracy and resolution for low range measurement. There are two types of ultrasonic transceivers, one relies on reflection from the surface, as the one used in [24,25]. The distance measurement of such ultrasonic sensor is the returned distance reflected from the ground, and the orientation of foot during walking is not considered. Therefore, it is not the vertical distance being measured. The other one is with ultrasonic transmitter and receiver on separate circuit boards using direct line of sight. The synchronization clock between transmitter and receiver is provided by an RF module [26,27]. There are only two receivers used in [26,27], which only measures one directional displacement, *i.e.*, displacement in the direction of progression.

In our paper, a wearable wireless ultrasonic sensor system for estimating 3-dimensional displacement to extract spatial-temporal gait parameters is developed. As compared with [26,27], the proposed system can measure not only the displacement in the direction of progression, but also the foot clearance, which occurs in the vertical direction and is an important parameter that is critical to the description of upright stability [5]. Additionally, the proposed ultrasonic motion analysis system is designed to allow patients to be monitored in an unconstrained environment. To reduce the usage of wires, we used the wireless sensor network concept with all the sensor nodes communicating to the coordinator wirelessly. Furthermore, the ultrasonic sensor node placed on human body is light and small. Additionally, the proposed motion analysis system is low-cost compared with the camera-based motion analysis system.

## 2. Related Work

The tracking techniques for locating a mobile device's position are studied using many approaches [28–30]. There are two major localization and tracking techniques, Receiver-Synchronization relative measurement (RS) and Global-Synchronization absolute range measurement (GS) [30]. RS range measurement only requires anchors to be synchronized and Time-Difference-of-Arrival (TDOA) technique is used for tracking and localization. In GS range measurement, both the mobile and the anchors are synchronized and the absolute distance can be estimated using Time-of-Arrival (TOA) technique. In our system, we prefer higher tracking and localization accuracy to accurately measure spatial-temporal gait parameters. Thus, we used the TOA-based tracking technique because the TDOA-based tracking technique has worse performance. RF signals are used in our system for synchronization between the anchors and the mobile. RF signal travels at the speed of light and the time it takes to reach mobile target is almost instantaneous and can be considered zero since the speed of ultrasound in air is much lower [31].

Under ideal range measurement case, an analytical localization method called trilateration, which uses only distance measurements, can be applied to identify the position of the mobile. For TOA-based localization technique, the target can be located at the intersecting point of several cycles that are formed by these anchors with known positions and distances to the mobile [31]. However, for a mobile target, it is not easy to track or localize because the range measurements are noisy and fluctuate, since the mobile can be located at anywhere in overlapped regions of such circles rather than being located at a single point at the intersection of the circles.

It is therefore desired to have accurate tracking and localization methods capable of filtering out the range measurement noises. One of the representative nonlinear state estimators is the least square (LS) method, which first transforms the nonlinear equations into linear ones and then solves the linear equations by LS-based estimator. Although the computation of this method is efficient, the tracking accuracy may not be sufficient [32]. Another typical method is proposed in [33], which begins with an initial guess and then applies least sum squared error to solve the nonlinear equation recursively. Although it provides better tracking performance, the initial guess should be carefully selected to guarantee the convergence of the iteration [34]. Therefore, many researchers proposed other methods to enhance the positioning performance. One representative implementation of indoor sensor network used to track a mobile is the Cricket of MIT [35,36]. It employs a hybrid approach involving the use of an Extended Kalman Filter (EKF) and Least Square Minimization to enhance the tracking and localization performance. EKF is the most commonly used nonlinear state estimator using the first or second order terms of the Taylor series expansion, which is most appropriate when the noise statistics is Gaussian distribution, to linearize the state and observation models [37]. Therefore, for some highly nonlinear dynamics, the linearization of EKF insufficiently characterizes the relationship. Therefore, we use Unscented Kalman Filter (UKF) to overcome such limitations of EKF, *i.e.*, the requirement for the noises to be Gaussian and the poor linearization of first or second order approximation. We will explain the tracking algorithm in more detail in the following section.

## 3. Methods

### 3.1. Ultrasonic Sensor System

**Figure 1.**
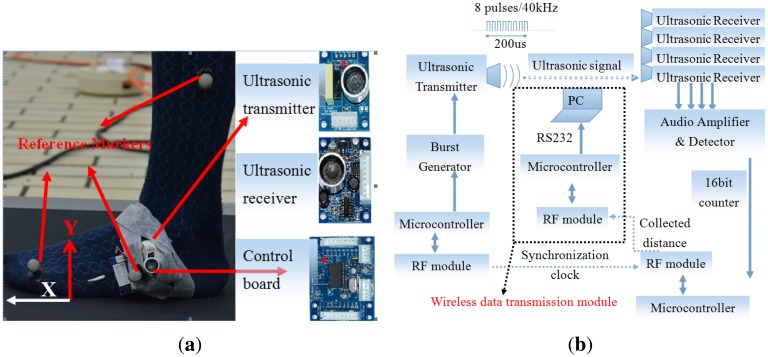
**(a)** Hardware system of the ultrasonic sensor system. The hardware comprises of a microcontroller and ultrasonic sensing components, which are on separate circuit boards. The ultrasonic transmitter is attached to the heel of subject with an elastic strap. **(b)** Block diagram of the ultrasonic motion analysis system.

The acquisition system that we developed for wearable gait analysis uses the wireless sensor network concept, with all mobile nodes communicating wirelessly with the coordinator to enable patients to be monitored in unconstrained environment, as shown in Figure 1. The proposed measurement system consists of one ultrasonic transmitter (referred to as the mobile with form factor: 4 cm × 3 cm × 1.6 cm) and four ultrasonic receivers (referred to as the anchors with the same form factor) made by Embedream studio, China [38]. The foot displacements measured using the TOA-based tracking technique were expressed in a global coordinate system that described foot position relative to the ground, as shown in Figure 1a. The X-axis was defined as the direction of progression, *i.e.*, anterior-posterior direction, and the Y-axis was defined as the vertical direction. The third axis of the coordinate system, *i.e.*, the Z-axis, was determined in such a way to form a right-handed coordinate system. However, for healthy subjects, the 2-dimensional model is sufficient to obtain spatial-temporal gait parameters, because the sagittal plane is the plane where the majority of movements take place.

Figure 1b shows the configuration of the ultrasonic measurement system. A battery-powered ultrasonic transmitter node is attached to the heel of the subject's foot. The mobile sensor node establishes communication with the coordinator node through a low power 430 MHz RF transceiver RFM12B. The coordinator node is also wirelessly communicating with the computer through a wireless data transmission module. The wireless data transmission module forwards all collected information to a personal computer through RS232 cable for postprocessing.

In the system, ultrasonic range measurements are initiated by a periodic trigger input with a pulse duration of 10 μs. Then, the ultrasound transmitter is triggered to produce an ultrasonic burst consisting of 8 pulses with a frequency of 40 kHz. Meanwhile, the RF module on the mobile node is triggered synchronously, thus sending out a data package with a timer starter command (TSC) using broadcast address to notify the anchors that an ultrasound signal has been transmitted. Once the anchor receives TSC, it will start its 16-bit counter to record the propagation delay from the mobile to the anchor. The transmission time of the RF signal from the mobile is negligible, since the speed of light is much faster than the speed of ultrasound. The 16-bit counter will stop counting immediately after each of the transmitted burst is detected by the anchor. Then the counted steps will be converted to propagation delay by multiplying the time resolution (instruction cycle) of the microcontroller. From this delay, the distance between the mobile and the anchor can be calculated by:

(1) $$d=t\cdot \upsilon_{s}$$

where *d* is the distance in meters, *t* is the propagation delay in seconds and *ν_s_* is the speed of ultrasound in air. The ultrasound velocity can be approximated to [26]:

(2) $$\upsilon_{s}=331.5+0.6T_{c}$$

where *T_c_* is the air temperature in degree Celsius. Together with the known positions of these anchors, the position of the mobile is located using the TOA-based tracking technique, which finds the intersection area of circles centered at each anchor with radius equal to the measured distances. The tracking algorithm is discussed in the following section.

### 3.2. Tracking Algorithm

In this section, we first explain how to establish a state space of nonlinear system to estimate the state of the moving target. Next we will apply UKF to enhance the performance of the tracking technique.

#### 3.2.1. Motion and Measurement Model

The mobile target in 3-dimensional field is represented by its position and velocity in X-Y-Z plane:

(3) $$X_{k}={\left[x_{k}\;y_{k}\;z_{k}\;{\dot{x}}_{k}\;{\dot{y}}_{k}\;{\dot{z}}_{k}\right]}^{T}$$

where *P_k_* = [*x_k_* *y_k_* *z_k_*]*^T^* are the position coordinates along X-, Y- and Z-axes at time step *k*, and ${\dot{P}}_{k}={\left[{\dot{x}}_{k}\;\dot{y}\;{\dot{z}}_{k}\right]}^{T}$ are the moving velocities with respect to these three axes at time step *k*. To formulate the dynamic transition process, the following state space equations are given

(4) $$\begin{array}{l} X_{k+1}=A_{k}X_{k}+G_{k}w_{k}\\ Y_{k}=h(X_{k})+\upsilon_{k} \end{array}$$

where

(5) $$A_{k}=\left[\begin{array}{llllll} 1 & 0 & 0 & \Delta t_{k} & 0 & 0\\ 0 & 1 & 0 & 0 & \Delta t_{k} & 0\\ 0 & 0 & 1 & 0 & 0 & \Delta t_{k}\\ 0 & 0 & 0 & 1 & 0 & 0\\ 0 & 0 & 0 & 0 & 1 & 0\\ 0 & 0 & 0 & 0 & 0 & 1 \end{array}\right],\;G_{k}=\left[\begin{array}{lll} \Delta t_{k}^{2}/2 & 0 & 0\\ 0 & \Delta t_{k}^{2}/2 & 0\\ 0 & 0 & \Delta t_{k}^{2}/2\\ \Delta t_{k} & 0 & 0\\ 0 & \Delta t_{k} & 0\\ 0 & 0 & \Delta t_{k} \end{array}\right]$$

where Δ*t_k_* = *t_k_*_+1_ − *t_k_* is the sampling interval. *w_k_* = [*w_x_w_y_* *w_z_*]*^T^* is the white Gaussian noise with zero mean and covariance matrix $W=diag(\sigma_{w_{x}}^{2},\sigma_{w_{y}}^{2},\sigma_{w_{z}}^{2})$. $V=diag(e_{1}^{2},e_{2}^{2},e_{3}^{2},e_{4}^{2})$ denotes the covariance matrix of the measurement noise *ν_k_*.

Let *d_ik_* denote the measured distance at the ith anchor using the equation:

(6) $$\begin{array}{c} d_{1k}=\sqrt{{\left(x_{k}-x_{1}\right)}^{2}+{\left(y_{k}-y_{1}\right)}^{2}+{\left(z_{k}-z_{1}\right)}^{2}}+e_{1k}\\ d_{2k}=\sqrt{{\left(x_{k}-x_{2}\right)}^{2}+{\left(y_{k}-y_{2}\right)}^{2}+{\left(z_{k}-z_{2}\right)}^{2}}+e_{2k}\\ d_{3k}=\sqrt{{\left(x_{k}-x_{3}\right)}^{2}+{\left(y_{k}-y_{3}\right)}^{2}+{\left(z_{k}-z_{3}\right)}^{2}}+e_{3k}\\ d_{4k}=\sqrt{{\left(x_{k}-x_{4}\right)}^{2}+{\left(y_{k}-y_{4}\right)}^{2}+{\left(z_{k}-z_{4}\right)}^{2}}+e_{4k} \end{array}$$

where [*x_i_* *y_i_* *z_i_*] is the known position of anchor *i*, *e_ik_* is the distance measurement error at anchor *i*, *Y_k_* = [*d*_1_ *_k_* *d*_2_*_k_* *d*_3_*_k_* *d*_4_*_k_*]*^T^*, and *ν_k_* = [*e*_1_*_k_* *e*_2_*_k_* *e*_3_*_k_* *e*_4_*_k_*]*^T^*.

#### 3.2.2. Unscented Kalman Filtering

The aforementioned state space model is a nonlinear dynamical system to the measurement distances and the state of foot motion. The approximation of UKF is to find a transformation that captures the mean and covariance of state random variable of length *n* through a nonlinear function [39]. We summarize the algorithm as follows.

For each time step *k*, start from ${\bar{X}}_{k/k}$ and *P_k_*_/_*_k_*,

1. Generate sigma points3. Update
  (7) $$\begin{array}{l} B_{k/k}=\sqrt{(n+\lambda )P_{k/k}}\\ \chi_{k/k}=\left[{\bar{X}}_{k/k}\;\;{\bar{X}}_{k/k}+B_{k/k}\;\;{\bar{X}}_{k/k}-B_{k/k}\right]\\ \in \;R_{n\times (2n+1)} \end{array}$$
2. Compute the a-priori statistics
  (8) $$\begin{array}{l} \chi_{k+1/k}^{*}=\chi_{k/k}\\ {\bar{X}}_{k+1/k}=\overset{2n}{\underset{i=0}{\text{∑}}}W_{i}^{m}\chi_{i,k+1/k}^{*}\\ P_{k+1/k}=G_{k}WG_{k}^{T}+\\ \overset{2n}{\underset{i=0}{\text{∑}}}W_{i}^{c}\left[\chi_{i,k+1/k}^{*}-{\bar{X}}_{k+1/k}\right]{\left[\chi_{i,k+1/k}^{*}-{\bar{X}}_{k+1/k}\right]}^{T} \end{array}$$
3. Update
  (9) $$\begin{array}{l} B_{k+1/k}=\sqrt{(n+\lambda )P_{k+1/k}}\\ \chi_{k+1/k}=\\ \left[{\bar{X}}_{k+1/k}\;\;{\bar{X}}_{k+1/k}+B_{k+1/k}\;\;{\bar{X}}_{k+1/k}-B_{k+1/k}\right]\\ Y_{k+1/k}=h(\chi_{k+1/k})\\ {\bar{Y}}_{k+1/k}=\overset{2n}{\underset{i=0}{\text{∑}}}W_{i}^{m}Y_{i,k+1/k} \end{array}$$
4. Compute the Kalman gain
  (10) $$\begin{array}{l} P_{yy}=\\ \overset{2n}{\underset{i=0}{\text{∑}}}W_{i}^{c}\left[Y_{i,k+1/k}-{\bar{Y}}_{k+1/k}\right]{\left[Y_{i,k+1/k}-{\bar{Y}}_{k+1/k}\right]}^{T}+V\\ P_{xy}=\overset{2n}{\underset{i=0}{\text{∑}}}W_{i}^{c}\left[X_{i,k+1/k}-{\bar{X}}_{k+1/k}\right]{\left[Y_{i,k+1/k}-{\bar{Y}}_{k+1/k}\right]}^{T}\\ K_{k+1}=P_{xy}P_{yy}^{-1} \end{array}$$
5. Compute the a-posteriori statistics
  (11) $$\begin{array}{l} {\bar{X}}_{k+1/k+1}={\bar{X}}_{k+1/k}+K_{k+1}\left(Y_{k+1}-{\bar{Y}}_{k+1/k}\right)\\ P_{k+1/k+1}=P_{k+1/k}+K_{k+1}P_{yy}K_{k+1}^{T} \end{array}$$
  *W* is the associated weight matrix:
  (12) $$\begin{array}{l} W_{0}^{m}=\lambda /(n+\lambda )\\ W_{0}^{c}=\lambda /(n+\lambda )+1-\alpha^{2}+\beta \\ W_{i}^{m}=1/(2(n+\lambda ))\\ W_{i}^{c}=1/(2(n+\lambda )),\;i=1,\ldots ,2n \end{array}$$

Parameter λ is the scaling factor, which is defined as:

(13) $$\lambda =\alpha^{2}(n+\kappa )-n$$

where *α* and *κ* control the spread of the sigma points around the mean of the state (α is usually set to a small positive value, e.g., 10^−3^) and *κ* is set to 0), *β* is related to the distribution of state variable (for Gaussian distribution, *β* = 2 is optimal).

### 3.3. Gait Parameters Estimation

#### 3.3.1. Autocorrelation Procedure

The idea of analyzing gait data by autocorrelation procedure is first proposed by Barrey *et al.* [40] and Auvinet *et al.* [41]. Then, the difference between biased and unbiased autocorrelation procedure for gait data analysis has been discussed by Moe *et al.* [9]. Here we summarize the autocorrelation procedure as follows.

The autocorrelation coefficient shows the degree of similarity between the given observations *a_i_* (*i* = 1,2,…,*N*) as a function of the time lag over successive time intervals, as given by:

(14) $$A=\sum_{i=1}^{N-m}a_{i}a_{i+m}$$

where *m* is the phase shift in the number of observations. The autocorrelation coefficients of a periodical signal will produce peak values for lag time equivalent to the cycle of the signal, which is the stride duration. Therefore, visual assessment of autocorrelation from the time series plot can be used to inspect the structure of a cyclic component.

As discussed in [9], either biased or unbiased autocorrelation coefficient can be computed for gait data analysis, but biased autocorrelation is not suitable for comparing autocorrelation coefficient over different time lags. The biased autocorrelation is the result of the raw autocorrelation coefficient *A* divided by the number of the observations in Equation (14):

(15) $$A_{biased}=\frac{1}{N}\sum_{i=1}^{N-m}a_{i}a_{i+m}$$

In Equation (15), the denominator *N* is the number of samples in observation *a_i_,* which is independent of the time lag *m*. It means that the number of samples in the numerator will decrease as the time lag *m* increases, and then the autocorrelation coefficient will attenuate. However, this is not the case in unbiased autocorrelation estimator, expressed as:

(16) $$A_{unbiased}=\frac{1}{N-m}\sum_{i=1}^{N-m}a_{i}a_{i+m}$$

Since the number of terms in the numerator *N* − *m* is always equal to the value of the denominator, there is no noticeable attenuation in the unbiased estimator.

**Figure 2.**
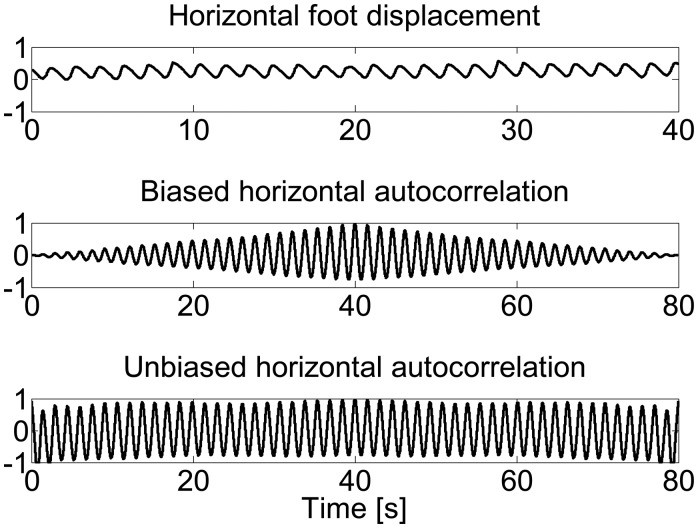
Horizontal foot displacement curve, biased and unbiased autocorrelation plots of normal gait.3.3.2. Estimation of Stride Regularity and Symmetry

Figure 2 shows the two different estimators for horizontal displacement during treadmill walking. The biased estimator shows clear periodicity but with attenuated amplitudes, while the unbiased estimator introduces no obvious attenuation except a deteriorated curve at the tails.

#### 3.3.2. Estimation of Stride Regularity and Symmetry

**Figure 3.**
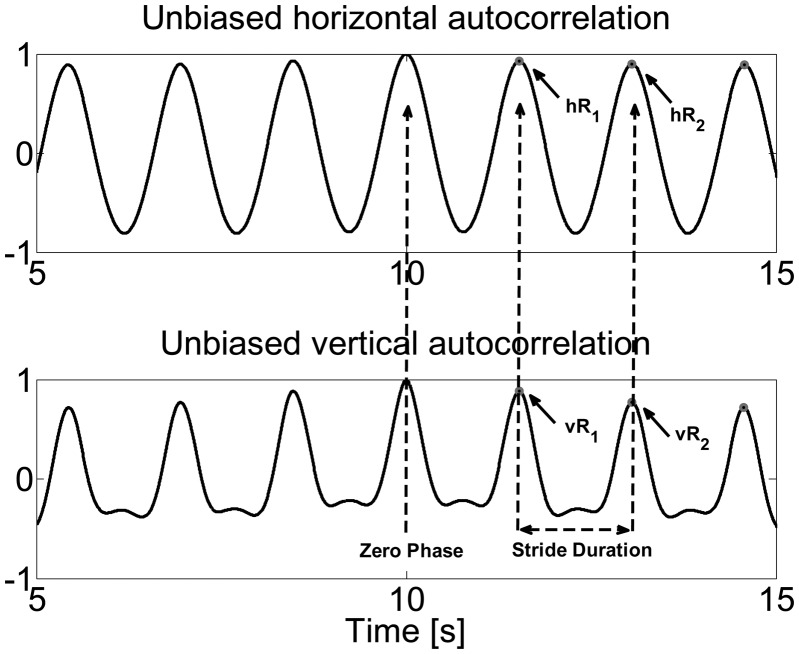
Horizontal and vertical unbiased autocorrelation plots of normal gait.

**Figure 4.**
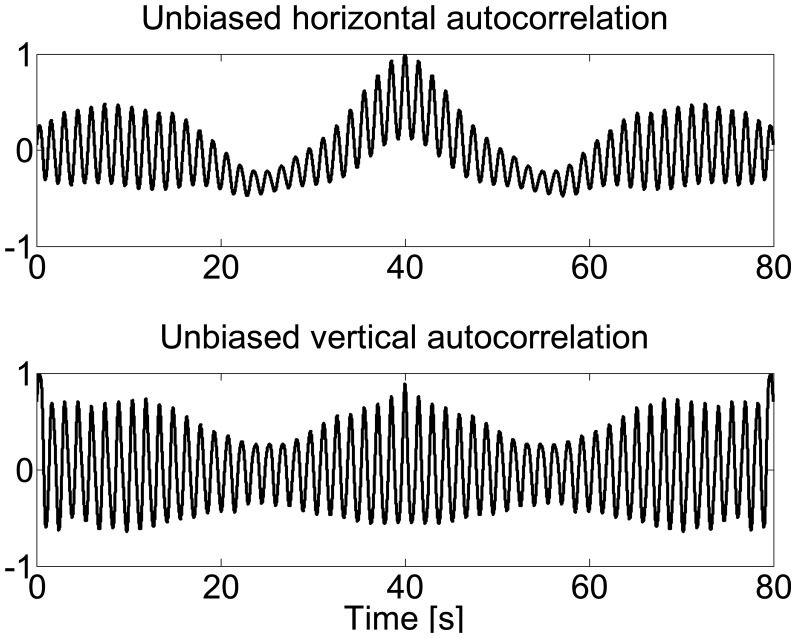
Horizontal and vertical unbiased autocorrelation plots of abnormal gait.

Figure 3 shows the normalized unbiased autocorrelation of horizontal and vertical foot displacement during treadmill walking. Since the first peak from the zero phase represents a phase shift of one stride duration, the autocorrelation coefficient at the periodic phase shift is defined as the regularity of the stride between neighboring strides, referred to as *hR_i_* for horizontal displacement and *νR_i_* for vertical displacement. Therefore, either for horizontal or vertical displacement, the closeness of *hR_i_*_+1_*/hR_i_* or *νR_i_*_+1_*/νR_i_* reflects the stride symmetry. Figure 4 demonstrates an example of asymmetric gait showing the unbiased autocorrelation sequence of the horizontal and vertical displacements.

#### 3.3.3. Estimation of Gait Parameters

From the estimated foot displacements by the proposed algorithm, the following spatial-temporal gait parameters can be obtained. With respect to the *j*th gait cycle, the estimators of the spatial-temporal gait parameters are as follows:

- Stride Length, *S*:
  (17) $$\begin{array}{l} S(j)=2St(j)\\ S(j)=Max(x_{j})-Min(x_{j}) \end{array}$$
  where the functions *Max*(*x*) and *Min*(*x*) return the maximum and minimum value of the variable *x*, and *x_j_* is the horizontal displacement in the *j*th gait cycle;
- Normalized Stride Length, *NS*:
  (18) NS(j)=S(j)/n
  where *NS* is defined as the stride length normalized by the number of strides *n*;
- Stride Duration, *T*:
  (19) $$T(j)=Index(max(x_{j+1}))-Index(max(x_{j}))$$
  where the function *Index*(*max*(*x_j_*)) returns the location of the maximum value in *x_j_*;
- Stride velocity, *V*:
  (20) V(j)=S(j)/T(j)
- Normalized Velocity, *NV*:
  (21) NV(j)=V(j)/n
  where the normalized speed is the speed as percentage of the number of strides *n*;
- Cadence, *C*:
  (22) C(j)=1/T(j)
  where the cadence is the number of strides in a second.

## 4. Experimental Validation

### 4.1. Experiment Setup

The proposed method was tested on 10 healthy subjects (age 25.7 ± 1.4 years, height 171.4 ± 6.5 cm, and weight 62.8 ± 5.6 kg) walking 5 min on a treadmill at slow, normal, and fast walking speeds, the results of which are presented in this paper. The subjects were recruited from students of Nanyang Technological University and none of them had a history of pathological gait disorders. To provide a more systematic validation, we conducted the experiments in a motion analysis lab with eight high speed cameras (Motion Analysis Eagle System, Santa Rosa, CA, USA) in the School of Mechanical and Aerospace Engineering at Nanyang Technological University. The Motion Analysis Eagle System consists of Eagle Digital Cameras and Cortex software, which captures complex 3D motion with extreme accuracy. System calibrations of the reference system should be done at both static (with 4-point calibration L-frame) and dynamic process (with 3-point calibration wand) to ensure an acceptable accuracy of the reference system. In our experiments, the accuracy of the reference system is 0.43 ± 0.18 mm (Average ± Standard deviation).

Figure 1a shows the placement of ultrasonic sensor and reflective markers on the test subject's foot. There were four anchors used in our experiment with positions *p*_1_ = [0 0 *0*]*^T^*, *p*_2_ = [0.324*m* 0 0]*^T^*, *p*_3_ = [0.324*m* 0.230*m* 0]*^T^*, *p*_4_ = [0 0.230*m* 0]*^T^*. The ultrasonic transmitter was attached to the heel of the foot pointing towards the four anchors, using elastic straps. In our method, only one ultrasonic sensor (transmitter) is needed to attach to the foot, which minimizes user discomfort and avoids complex calibration procedures and synchronization issues. All data transmission between anchors, coordinator and transmitter are done wirelessly through the RF module. Therefore, it does not restrict the movement of subjects. The ultrasonic sensor data were acquired at 50 Hz. Data from the reference system were captured at 200 Hz. The difference between the sampling rate of these two systems was compensated by linear interpolation. All data were low-pass filtered by second order low-pass Butterworth filter at 10 Hz.

### 4.2. Processing of Measured Data

In order to compare the estimated spatial-temporal gait parameters at each recorded gait cycle, the foot trajectory estimate with proposed ultrasonic sensors was temporally delayed to match the trajectory estimated by the camera reference system, by finding the maximum values of cross-correlation between these two trajectories. To quantify the performance of the proposed system against the camera reference system, the mean and standard deviation (std) were calculated on the datasets of difference, as well as the Root Mean Square Error (RMSE). This is followed by using the analysis of variance (ANOVA) to test differences in the means of the ten subjects for statistical significance. Finally, walking speed was estimated using the proposed ultrasonic sensor configuration to check significant changes over different speeds. Two-sample *t*-tests were performed on the walking velocity and the extracted gait parameters to assess the significance of change in these gait parameters with speed, and thus investigate the effect of walking velocity on the difference between the proposed system and the reference system in gait parameters estimation.

### 4.3. Parameters Identification

As the system modelling we have adopted in Section 3.2.1., the process and measurement noise statistics should be estimated. A wooden pendulum was constructed using a uniaxial pivot so that it swung through an arc [42]. The ultrasonic transmitter was placed at the end of this pendulum, and a reflective marker was also located approximately in alignment with the ultrasonic transmitter head. The pendulum was raised up at an angle and allowed to drop freely until it came to a stable position. This action was repeated *M* times. The experiment helps to find suitable values of process noise *W* and measurement noise *V*. The measurements from camera system, *r_i_*, are referred to as the actual distance for test *i*, and there are *N* measurement samples $m_{i}^{j}$ collected for each test, where *j* = 1, …, *N*.

#### 4.3.1. Process Noise Statistics in Kalman Filter

As the process noise in UKF is an independent variable, it is difficult to get an exact value [31]. Here, we consider it as a velocity noise in X, Y and Z directions in mm/s. The process noise *W* was estimated using numerical methods. By varying the values of *σ* *_wx_*, *σ* *_wy_* and *σ* *_wz_*, we will get the corresponding trajectory of the mobile to compute the RMSE value. Typical values of *σ* *_wx_*, *σ* *_wy_* and *σ* *_wz_* will be selected when their corresponding RMSE is minimal. The typical values of *W* used in our experiments are *σ* *_wy_* = 30, *σ* *_wy_* =25, *σ* *_wy_* = 10.

#### 4.3.2. Measurement Noise Statistics in Kalman Filter

It is reasonable to assume that all anchors have independent distributed noise. Then, the mean and covariance of the measurement noise can be evaluated by the pendulum experiments. Using the data obtained from the specific experiments, straightforward calculations lead to the estimation of mean and variance of the measurement errors

(23) $$\begin{array}{l} u=\frac{1}{MN}\overset{M}{\underset{i=1}{\text{∑}}}\overset{N}{\underset{j=1}{\text{∑}}}\left(m_{i}^{j}-r_{i}\right)\\ e^{2}=\frac{1}{M(N-1)}\overset{M}{\underset{i=1}{\text{∑}}}\overset{N}{\underset{j\text{=}1}{\text{∑}}}{\left(m_{i}^{j}-u\right)}^{2} \end{array}$$

Typical value of *V* used in our experiments is *V* = *diag*(11, 9.3,9, 9) with the units as mm^2^. In other words, the accuracy of distance measurement by each ultrasonic sensors is around 3 mm.

The results of pendulum experiment have been shown in Table 1. The Net RMSE is defined as $Net\;RMSE=\sqrt{X_{RMSE}^{2}+Y_{RMSE}^{2}+Z_{RMSE}^{2}}$. The difference between the two systems was obtained with an RMSE value of 4.08 mm in horizontal direction (X), 0.72 mm in vertical direction (Y) and 1.08 mm in lateral direction. The Net RMSE value of 4.28 mm in 3D space of UKF estimator is achievable in the pendulum model.

### 4.4. Results

#### 4.4.1. Performance Comparison

The mean and standard deviation in stride length, stride duration, and stride velocity estimation between the proposed system and the reference system together with RMSE value are reported in Tables 2 and 4 for all subjects walking at normal speed. On average, across all subjects, the estimates of stride length from the proposed method were 0.001 m less than the reference measurements. The overall RMSE value is about 0.027 m, which is 2.3% of the mean estimated stride length of the reference system. The mean and standard deviation of stride duration at normal walking speed is reported as 1.18 ± 0.02 s by the reference system and 1.18 ± 0.04 s by the proposed system, which shows no mean difference between the two systems. The average error across all subjects of RMSE of the estimated stride duration is 0.035 s with 3% percent error. The mean and standard deviation in the estimation of the stride velocity is reported in Table 4, which shows that the proposed method slightly overestimated the stride velocity by 0.001 m/s with an RMSE value of 0.036 m/s, occupying 3.6% of the proposed estimates of stride velocity.

We have elaborated how gait cycle periodicity of foot displacement data can be used to extract stride regularity and symmetry by unbiased autocorrelation procedure in Section 3.3.2. Tables 5 and 6 show the mean and standard deviation of the reference system and the proposed system together with RMSE values in detecting horizontal and vertical stride symmetry respectively for each subject. The mean and standard deviation data of horizontal stride symmetry are 1.001 ± 0.021 by the reference system and 0.999 ± 0.027 by the proposed system, which shows that the ultrasonic-based horizontal stride symmetry was underestimated by a negligible error of 0.002. An RMSE of 0.013 with 1.3% percent error is also reported for the estimates of horizontal stride symmetry across all subjects. In the contrary, the ultrasonic-based vertical stride symmetry was overestimated by 0.007, where the RMSE value is 0.034 with a percent error of 3.5%. In summary, all the numerical results show a clinically acceptable accuracy of the proposed system with an average percent error of 2.7% for all the estimated gait parameters.

#### 4.4.2. Statistical Analysis

**Figure A1.**
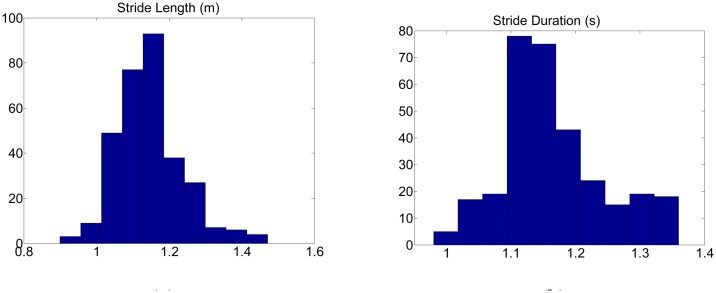

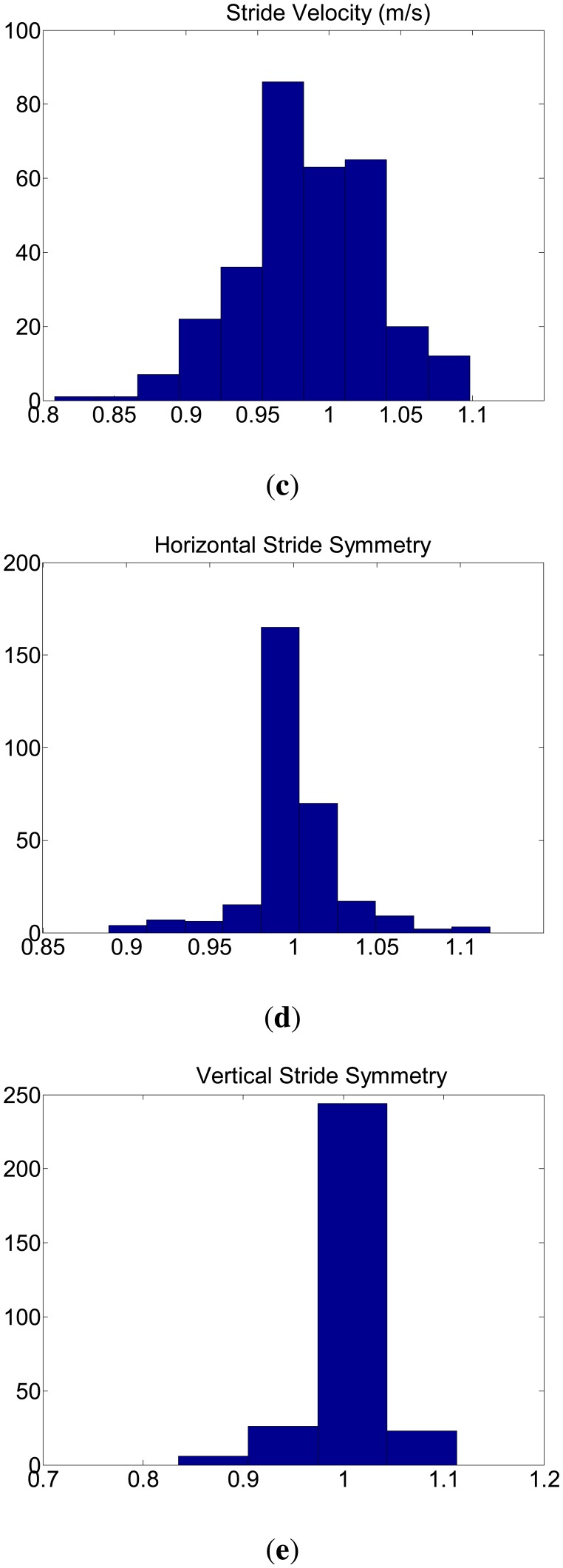
Histograms of all estimates of stride length, stride duration, stride velocity, horizontal stride symmetry and vertical stride symmetry.15452

In this part, ANOVA has been performed to test differences in the means (for ten subjects) for statistical significance. We base this test on a comparison of the variance due to the between-groups variability (called Mean Square Effect, or *MS_effect_*) with the variance due to the within-group variability (called Mean Square Error, or *MS_error_*). Before applying ANOVA, whether the distribution of the data is normal or not should be checked. Results are reported in Table 7 and Figure A1. In Table 7, *H* = *0* indicates that the null hypothesis (“mean is zero”) cannot be rejected at the *5*% significance level. The p-value is the probability of observing the given result by chance if the null hypothesis is true. Large value of p shows the validity of the null hypothesis. As in Table 7, not only all values of *H* are equal to zero and the values of *p* are equal to one, but also the means of estimates are located in the 95% confidence interval. Therefore, the estimated parameters are normally distributed.

**Figure B1.**
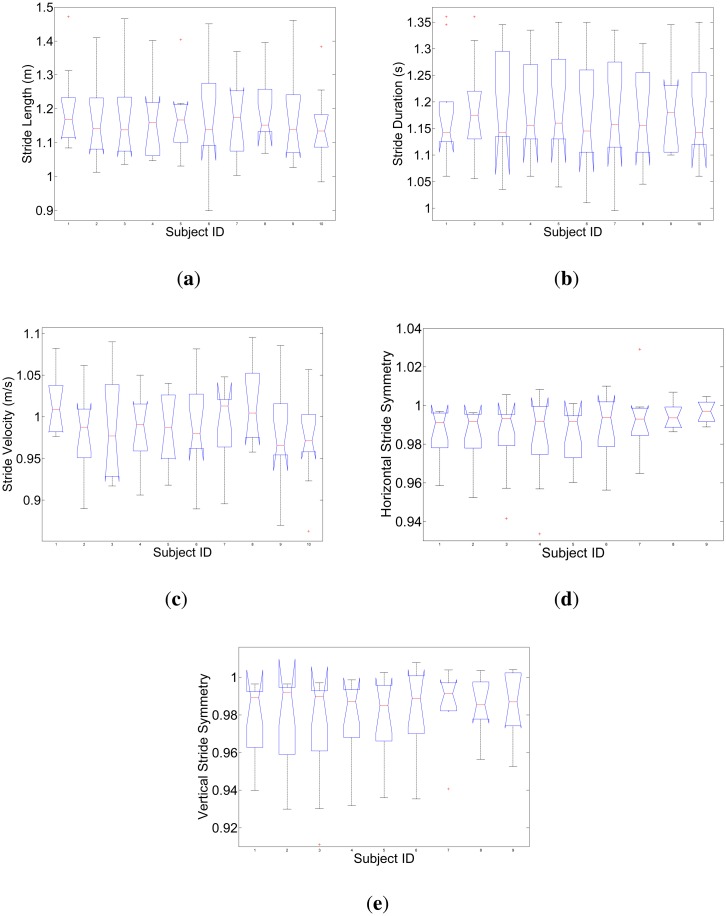
Boxplots of stride length, stride duration, stride velocity, horizontal stride symmetry and vertical stride symmetry for each subject.

Under the null hypothesis (that there are no mean differences among subjects), we compare the *MS_effect_* and *MS_error_* via the *F*-test, which tests whether the ratio of the two variance estimates is significantly greater than 1. Otherwise, we will accept the null hypothesis of no differences between the means, *i.e.*, the means (in the population) are not statistically different from each other. Figure B1 shows the boxplots of stride length, stride duration, stride velocity, horizontal stride symmetry and vertical stride symmetry for each subject. The analysis of variance is summarized in Table C1. As shown in Table C1, for all estimated gait parameters, the small value of between-groups sum of squares likely indicates no differences among the subjects. Additionally, the values of F are less than 1, which indicates that the means of all gait parameters are not statistically different.

#### 4.4.3. The Effect of Walking Speed on the Measurement of Gait parameters

Table 8 provides the numerical results of estimated gait parameters by the proposed system compared with those obtained from the reference system using the pair *t*-test. Significant difference was assumed when the null hypothesis can be rejected at *p*-value smaller than 0.05. The walking speed, on average, across all subjects was significantly different (*p* < 0.001 for the two measurement systems) among slow (0.54 ± 0.02 m/s), normal (0.99 ± 0.04 m/s)and fast (1.40 ± 0.04 m/s) speed. The influence of walking speed on all spatial-temporal gait parameters was tested by the mean and standard deviation values for the proposed and reference systems.

**Figure 5.**
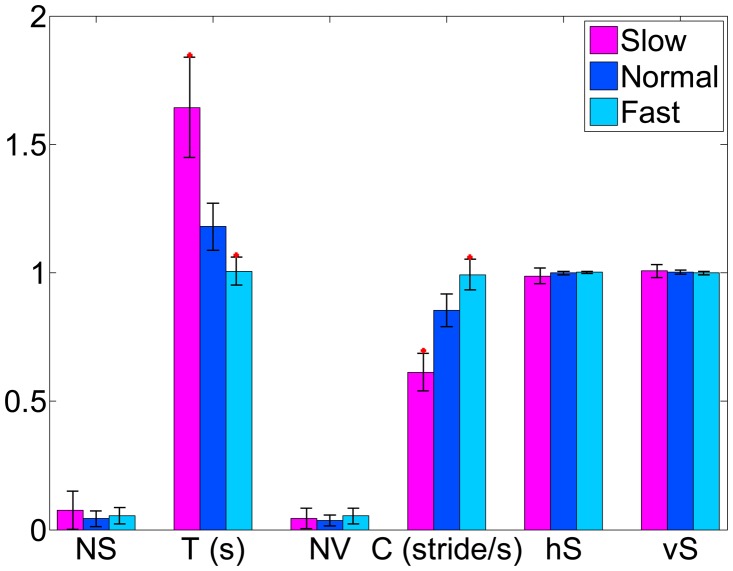
The effect of walking speeds on spatial-temporal gait parameters at slow, normal and fast walking speed, where * indicates the significant differences with normal speed (*p* < 0.05).

The measurement errors of estimated S, NS, NV, C, and vS were not affected significantly by the changes in walking velocity (*p* > 0.05). Particularly, there is no difference in cadence estimation between the proposed and reference systems. The influence of speed on the measurement errors of stride duration T was found to be significantly higher (*p* < 0.05) at fast speed, but it was not significant for V and hS. This can be interpreted as the lower temporal resolution at higher walking speed. Figure 5 shows significant changes in T and C, but there is no significant change in other parameters. Although the means of both horizontal stride symmetry and vertical stride symmetry are not statistically significant, the largest variations at slow speeds were observed. Therefore, the stride symmetry can be used as warning sign of walking disorders.

## 5. Discussion and Conclusions

In this paper, a low-cost ultrasonic motion analysis system using an ultrasonic transmitter and four receivers to track the foot displacement in 3D space is developed. The proposed motion analysis system has been validated against camera-based system with 10 healthy subjects, and shown to produce accurate estimates of some spatial-temporal gait parameters including stride length with RMSE value of 0.027 m (2.3%), stride duration with RMSE value of 0.035 s (3%), stride velocity with RMSE value of 0.036 m/s (3.6%), horizontal stride symmetry with RMSE value of 0.013 (1.3%) and vertical stride symmetry with RMSE value of 0.034 m (3.5%). We have further evaluated the influence of walking speed on these gait parameters by paired *t*-test.

The proposed system includes some ultrasonic sensors and micro-controllers, estimated today at about a cost of $100, which is inexpensive compared with current commercial camera-based system. With the rapid development of technology, the performance of these sensors will continue to improve while becoming available at even lower price. Therefore, low-cost in-home monitoring system for clinical applications can be possible.

As the work stated here is a first step to evaluate the feasibility of the proposed ultrasonic system, only ten healthy subjects participated in the experiments and were instructed to walk 5 min on treadmill at different speeds. The walking experiments were chosen on treadmill due to the limited measurement volume of the reference camera-based system. In addition, we can get a cyclic signal on horizontal displacement to analyze the stride symmetry. Although the proposed ultrasonic motion analysis system also has such limitations, the maximum propagation distance of the ultrasonic signal used in our system is 20 m, which is large enough for indoor applications.

Although the positive results showed the feasibility of applying such a system for in-home monitoring, there is an issue to be addressed in further research, *i.e.*, how to deal with the multipath propagation. All the experiments in this study are conducted under line-of-sight condition, where the ultrasonic transmitter faces all the receivers without any obstacles between them. Therefore, for 3D displacements, according to spherical positioning technique, a minimum of 4 anchors with known positions are required. The method used in our experiment to mitigate the multipath propagation is by setting an inhibit time, *i.e.*, the ultrasound detector will be disabled within the inhibit time to detect an ultrasound signal, and will be enabled again after the inhibit time has passed. Another possible solution is that we can use more receivers, which can not only account for multipath propagation, but also increase the measurement volume and accuracy of the proposed system [34].

Long-term monitoring is expected to be more challenging as demonstrated in some studies [18,43]. In [18,43], foot clearance measurement using inertial sensors is proposed and investigated. The displacement estimation requires double integration of measured accelerations from inertial sensors, which involves error accumulation over long time monitoring. Even though the growth uncertainty that arises from the integration of acceleration error can be mitigated by periodic corrections like ZUPT, the prerequisite is that the initial and/or terminal contact should be detected correctly, but it may be difficult in some type of abnormal gait. Although not specifically studied under long term monitoring, the proposed system does not have significant error accumulation for a 5 min walk.

In summary, we used a low-cost ultrasonic motion analysis system to extract spatial-temporal gait parameters, and tested the feasibility of the system against a reference camera-based system. The positive results demonstrated a great potential in using this low-cost system for clinical applications such as rehabilitation, gait analysis, and sports. For further work, experiments conducted with patients in collaboration with a hospital are being planned using our system.

## Figures and Tables

**Table C1.** Summary of the analysis of variance of stride length, stride duration, stride velocity, horizontal stride symmetry, and vertical stride symmetry.

**ANOVA Table: Stride Length**					
**Source of Variation**	**Sum of Squares**	**Degrees of Freedom**	**Mean Square**	**F**	**Prob > F**
Between groups	0.01804	9	0.002	0.14	0.9984
Within groups	1.29563	90	0.0144		
Total	1.31367	99			

**ANOVA Table: Stride Duration**					

**Source of Variation**	**Sum of Squares**	**Degrees of Freedom**	**Mean Square**	**F**	**Prob > F**

Between groups	0.00446	9	0.0005	0.05	1
Within groups	0.81343	90	0.00904		
Total	0.81789	99			

**ANOVA Table: Stride Velocity**					

**Source of Variation**	**Sum of Squares**	**Degrees of Freedom**	**Mean Square**	**F**	**Prob > F**

Between groups	0.01709	9	0.0019	0.72	0.6936
Within groups	0.23895	90	0.00266		
Total	0.25604	99			

**ANOVA Table: Horizontal Stride Symmetry**					

**Source of Variation**	**Sum of Squares**	**Degrees of Freedom**	**Mean Square**	**F**	**Prob > F**

Between groups	0.00069	9	0.00009	0.13	0.9976
Within groups	0.05293	90	0.00059		
Total	0.05363	99			

**ANOVA Table: Vertical Stride Symmetry**					

**Source of Variation**	**Sum of Squares**	**Degrees of Freedom**	**Mean Square**	**F**	**Prob > F**

Between groups	0.00047	9	0.00005	0.06	0.9999
Within groups	0.0793	90	0.00088		
Total	0.07977	99			

**Table 1.** Errors of pendulum experiment in 3D space compared with motion capture system.

	**Mean (mm)**	**std (mm)**	**RMSE (mm)**	**Net RMSE (mm)**
X	0.02	4.08	4.08	4.08
Y	0.03	0.72	0.72	4.14
Z	0.09	1.08	1.08	4.28

**Table 2.** Mean and standard deviation (in meters) of the reference (Ref) and proposed (Pro) systems and RMSE in detecting stride length for each subject. Averaged values across the ten subjects are also reported.

	**Subject**	**1**	**2**	**3**	**4**	**5**	**6**	**7**	**8**	**9**	**10**	**Average**	**Peak**
**Ref**	**mean**	1.147	1.071	1.158	1.421	1.117	1.137	1.101	1.276	1.041	1.224	**1.169**	**1.421**
	**std**	0.047	0.019	0.019	0.037	0.046	0.056	0.041	0.035	0.055	0.034	**0.039**	**0.056**

**Pro**	**mean**	1.147	1.070	1.157	1.420	1.116	1.136	1.100	1.274	1.040	1.223	**1.168**	**1.420**
	**std**	0.057	0.025	0.027	0.057	0.050	0.064	0.034	0.038	0.067	0.035	**0.045**	**0.067**

	**RMSE**	0.029	0.022	0.013	0.033	0.029	0.024	0.020	0.029	0.042	0.030	**0.027**	**0.042**

**Table 3.** Mean and standard deviation (in seconds) of the reference (Ref) and proposed (Pro) systems and RMSE in detecting stride duration for each subject. Averaged values across the ten subjects are also reported.

	**Subject**	**1**	**2**	**3**	**4**	**5**	**6**	**7**	**8**	**9**	**10**	**Average**	**Peak**
**Ref**	**mean**	1.237	1.109	1.134	1.344	1.160	1.155	1.114	1.309	1.050	1.192	**1.180**	**1.344**
	**std**	0.031	0.011	0.014	0.015	0.021	0.025	0.020	0.020	0.030	0.014	**0.020**	**0.031**

**Pro**	**mean**	1.236	1.108	1.134	1.341	1.161	1.155	1.114	1.308	1.051	1.190	**1.180**	**1.341**
	**std**	0.053	0.031	0.033	0.024	0.049	0.039	0.044	0.045	0.050	0.046	**0.041**	**0.053**

	**RMSE**	0.042	0.030	0.027	0.026	0.046	0.029	0.034	0.035	0.033	0.046	**0.035**	**0.046**

**Table 4.** Mean and standard deviation (in meters per second) of the reference (Ref) and proposed (Pro) systems and RMSE in detecting stride velocity for each subject. Averaged values across the ten subjects are also reported.

	**Subject**	**1**	**2**	**3**	**4**	**5**	**6**	**7**	**8**	**9**	**10**	**Average**	**Peak**
**Ref**	**mean**	0.927	0.966	1.021	1.057	0.964	0.984	0.989	0.974	0.992	1.027	**0.990**	**1.057**
	**std**	0.032	0.017	0.011	0.025	0.039	0.043	0.043	0.028	0.049	0.034	**0.032**	**0.049**

**Pro**	**mean**	0.928	0.966	1.021	1.060	0.962	0.984	0.989	0.975	0.991	1.029	**0.991**	**1.060**
	**std**	0.042	0.032	0.027	0.042	0.049	0.053	0.046	0.038	0.064	0.053	**0.045**	**0.064**

	**RMSE**	0.038	0.033	0.024	0.021	0.049	0.032	0.035	0.031	0.047	0.053	**0.036**	**0.053**

**Table 5.** Mean and standard deviation of the reference (Ref) and proposed (Pro) systems and RMSE in detecting horizontal stride symmetry (hS) for each subject. Averaged values across the ten subjects are also reported.

	**Subject**	**1**	**2**	**3**	**4**	**5**	**6**	**7**	**8**	**9**	**10**	**Average**	**Peak**
**Ref**	**mean**	1.002	1.003	1.000	1.012	1.002	0.996	1.000	0.995	0.999	1.000	**1.001**	**1.012**
	**std**	0.021	0.010	0.007	0.021	0.016	0.033	0.006	0.014	0.067	0.009	**0.021**	**0.067**

**Pro**	**mean**	1.004	1.001	1.001	1.011	1.001	0.991	1.000	0.996	0.989	1.000	**0.999**	**1.011**
	**std**	0.027	0.007	0.008	0.023	0.022	0.049	0.013	0.016	0.089	0.018	**0.027**	**0.089**

	**RMSE**	0.010	0.004	0.004	0.013	0.011	0.021	0.009	0.008	0.038	0.010	**0.013**	**0.038**

**Table 6.** Mean and standard deviation of the reference (Ref) and proposed (Pro) systems and RMSE in detecting vertical stride symmetry (vS) for each subject. Averaged values across the ten subjects are also reported.

	**Subject**	**1**	**2**	**3**	**4**	**5**	**6**	**7**	**8**	**9**	**10**	**Average**	**Peak**
**Ref**	**mean**	1.012	1.000	0.995	0.931	0.996	1.002	1.002	1.004	1.000	1.007	**0.995**	**1.012**
	**std**	0.045	0.009	0.019	0.181	0.016	0.051	0.010	0.018	0.065	0.019	**0.043**	**0.181**

**Pro**	**mean**	1.009	1.002	0.997	0.991	0.996	1.003	1.002	1.012	0.997	1.011	**1.002**	**1.012**
	**std**	0.042	0.038	0.017	0.069	0.038	0.038	0.022	0.044	0.088	0.030	**0.043**	**0.088**

	**RMSE**	0.017	0.036	0.023	0.079	0.028	0.031	0.020	0.039	0.048	0.023	**0.034**	**0.079**

**Table 7.** Normality test of gait parameters.

		**Mean**		**std**		**H**		**p**		**95% Confidence Interval**				
**S(m)**		1.147		0.093		0.000		1.000		1.137		1.158		
**T (s)**		1.164		0.080		0.000		1.000		1.155		1.173	
**V (m/s)**		0.986		0.046		0.000		1.000		0.980		0.991	
**hS**		1.000		0.028		0.000		1.000		0.997		1.003		
**vS**		0.999		0.048		0.000		1.000		0.994		1.005		

**Table 8.** Foot parameters at different walking velocities, * indicates the significant differences with normal speed (*p* < 0.05).

**Speed**		**Slow**			**Normal**		**Fast**		
		
		**Mean**	**std**	**p**	**Mean**	**std**	**Mean**	**std**	**p**
**S(m)**	Ref	0.884	0.095	0.000 *	1.169	0.112	1.410	0.091	0.000 *
	Pro	0.884	0.095	0.000 *	1.168	0.112	1.409	0.090	0.000 *
	RMSE	0.024	0.012	0.623	0.027	0.008	0.031	0.021	0.550

**NS**	Ref	0.075	0.074	0.329	0.043	0.031	0.054	0.031	0.425
	Pro	0.075	0.074	0.329	0.043	0.031	0.054	0.031	0.425
	RMSE	0.001	0.001	0.339	0.001	0.001	0.001	0.001	0.547

**T(s)**	Ref	1.645	0.192	0.000 *	1.180	0.092	1.006	0.055	0.000 *
	Pro	1.644	0.195	0.000 *	1.180	0.091	1.006	0.054	0.000 *
	RMSE	0.039	0.015	0.592	0.035	0.007	0.027	0.005	0.019 *

**V(m/s)**	Ref	0.539	0.023	0.000 *	0.990	0.037	1.402	0.044	0.000 *
	Pro	0.539	0.024	0.000 *	0.991	0.038	1.402	0.043	0.000 *
	RMSE	0.020	0.008	0.005 *	0.036	0.010	0.048	0.017	0.073

**NV**	Ref	0.044	0.040	0.692	0.035	0.021	0.053	0.030	0.137
	Pro	0.044	0.040	0.695	0.035	0.022	0.053	0.030	0.138
	RMSE	0.001	0.001	0.693	0.001	0.000	0.002	0.001	0.131

**C(stride/s)**	Ref	0.614	0.073	0.000 *	0.853	0.063	0.993	0.059	0.000 *
	Pro	0.614	0.073	0.000 *	0.853	0.063	0.993	0.059	0.000 *
	RMSE	0.000	0.000	NaN	0.000	0.000	0.000	0.000	NaN

**hS**	Ref	0.993	0.025	0.368	1.001	0.004	1.001	0.003	0.996
	Pro	0.988	0.031	0.267	0.999	0.006	1.002	0.005	0.334
	RMSE	0.024	0.016	0.039 *	0.013	0.010	0.016	0.015	0.587

**vS**	Ref	1.021	0.040	0.124	0.995	0.023	1.002	0.006	0.346
	Pro	1.007	0.025	0.652	1.002	0.007	0.999	0.007	0.375
	RMSE	0.105	0.111	0.090	0.034	0.019	0.027	0.016	0.333

## References

[1] Liu T., Inoue Y., Shibata K. (2010). A wearable ground reaction force sensor system and its application to the measurement of extrinsic gait variability. Sensors.

[2] Dadashi F., Mariani B., Rochat S., Biila C.J., Santos-Eggimann B., Aminian K. (2013). Gait and foot clearance parameters obtained using shoe-worn inertial sensors in a large-population sample of older adults. Sensors.

[3] Martin Schepers H., van Asseldonk E.H., Baten C., Veltink P.H. (2010). Ambulatory estimation of foot placement during walking using inertial sensors. J. Biomech..

[4] Salarian A., Russmann H., Vingerhoets F.J., Dehollaini C., Blanc Y., Burkhard P.R., Aminian K. (2004). Gait assessment in Parkinson's disease: Toward an ambulatory system for long-term monitoring. IEEE Trans. Biomed. Eng..

[5] Khandoker A.H., Taylor S.B., Karmakar C.K., Begg R.K., Palaniswami M. (2008). Investigating scale invariant dynamics in minimum toe clearance variability of the young and elderly during treadmill walking. IEEE Trans. Neural Syst. Rehabil. Eng..

[6] Begg R., Best R., Dell'Oro L., Taylor S. (2007). Minimum foot clearance during walking: Strategies for the minimisation of trip-related falls. Gait Posture.

[7] Lai D.T., Taylor S.B., Begg R.K. (2012). Prediction of foot clearance parameters as a precursor to forecasting the risk of tripping and falling. Hum. Mov. Sci..

[8] Allen J.L., Kautz S.A., Neptune R.R. (2011). Step length asymmetry is representative of compensatory mechanisms used in post-stroke hemiparetic walking. Gait Posture.

[9] Moe-Nilssen R., Helbostad J.L. (2004). Estimation of gait cycle characteristics by trunk accelerometry. J. Biomech..

[10] Kose A., Cereatti A., DellaCroce U. (2012). Bilateral step length estimation using a single inertial measurement unit attached to the pelvis. J. Neuroeng. Rehabil..

[11] Tao W., Liu T., Zheng R., Feng H. (2012). Gait analysis using wearable sensors. Sensors.

[12] Lee G.X., Low K.S. (2012). A Factorized Quaternion Approach to Determine the Arm Motions Using Triaxial Accelerometers With Anatomical and Sensor Constraints. IEEE Trans. Instrum. Meas..

[13] Qi Y., Soh C.B., Gunawan E., Low K.S., Maskooki A. (2014). A Novel Approach to Joint Flexion/Extension Angles Measurement Based on Wearable UWB Radios. IEEE J. Biomed. Health Inform..

[14] Qi Y., Soh C.B., Gunawan E., Low K.S., Maskooki A. Measurement of knee flexion/extension angle using wearable UWB radios.

[15] Schepers H.M., Koopman H., Veltink P.H. (2007). Ambulatory assessment of ankle and foot dynamics. IEEE Trans. Biomed. Eng..

[16] Schepers H.M., van Asseldonk E.H., Buurke J.H., Veltink P.H. (2009). Ambulatory estimation of center of mass displacement during walking. IEEE Trans. Biomed. Eng..

[17] Lee M.K., Le N.S., Fang A.C., Koh M.T. (2009). Measurement of body segment parameters using dual energy X-ray absorptiometry and three-dimensional geometry: An application in gait analysis. J. Biomech..

[18] Lai D., Begg R., Charry E., Palaniswami M., Hill K. Measuring toe clearance using a wireless inertial sensing device.

[19] Petruska A.J., Meek S.G. Non-drifting limb angle measurement relative to the gravitational vector during dynamic motions using accelerometers and rate gyros.

[20] Panahandeh G., Mohammadiha N., Leijon A., Handel P. (2013). Continuous hidden Markov model for pedestrian activity classification and gait analysis. IEEE Trans. Instrum. Meas..

[21] Ruiz A.R.J., Granja F.S., Prieto Honorato J.C., Rosas J.I.G. (2012). Accurate pedestrian indoor navigation by tightly coupling foot-mounted IMU and RFID measurements. IEEE Trans. Instrum. Meas..

[22] Corrales J.A., Candelas F., Torres F. Hybrid tracking of human operators using IMU/UWB data fusion by a Kalman filter.

[23] Qi Y., Soh C.B., Gunawan E., Low K.S., Maskooki A. Using wearable UWB radios to measure foot clearance during walking.

[24] Wahab Y., Bakar N.A. Gait analysis measurement for sport application based on ultrasonic system.

[25] Wahab Y., Zayegh A., Bakar N. (2012). Foot-to-ground Clearance Measurement: Analysis and Modelling of MEMS Applicability. Int. J. Res. Rev. Signal Acquis. Process. (IJRRSAP).

[26] Huitema R.B., Hof A.L., Postema K. (2002). Ultrasonic motion analysis system: measurement of temporal and spatial gait parameters. J. Biomech..

[27] Maki H., Ogawa H., Yonezawa Y., Hahn A.W., Caldwell W.M. (2012). A new ultrasonic stride length measuring system. Biomed. Sci. Instrum..

[28] Lee S., Lee W., You K. (2009). TDoA Based UAV Localization Using Dual-EKF Algorithm. In *Control and Automation;*.

[29] Chen Z.X., Wei H.W., Wan Q., Ye S.F., Yang W.L. (2009). A supplement to multidimensional scaling framework for mobile location: A unified view. IEEE Trans. Signal Process.

[30] Zhou Y., Law C., Guan Y., Chin F. (2011). Indoor elliptical localization based on asynchronous UWB range measurement. IEEE Trans. Instrum. Meas..

[31] Shareef A., Zhu Y. (2009). Localization using extended Kalman filters in wireless sensor networks. Graduate Student Scholarly and Creative Submissions.

[32] Zhou Y., Law C.L., Guan Y.L., Chin F. Localization of passive target based on UWB backscattering range measurement.

[33] Foy W. (1976). Position-location solutions by Taylor-series estimation. IEEE Trans. Aerosp. Electr. Syst..

[34] Qi Y., Soh C.B., Gunawan E., Low K.S., Maskooki A. An Accurate 3D UWB Hyperbolic Localization in Indoor Multipath Environment Using Iterative Taylor-Series Estimation.

[35] Priyantha N.B. (2005). The Cricket Indoor Location System. Ph.D. Thesis.

[36] Smith A., Balakrishnan H., Goraczko M., Priyantha N. Tracking moving devices with the cricket location system.

[37] Hartikainen J., Solin A., Sarkka S. (2011). Optimal Filtering with Kalman Filters and Smoothers.

[38] Qi Y., Soh C.B., Gunawan E., Low K.S. (2014). Ambulatory Measurement of 3-Dimensional Foot Displacement During Treadmill Walking Using Wearable Wireless Ultrasonic Sensor Network. IEEE J. Biomed. Health Inform..

[39] Bando M., Kawamata Y., Aoki T. (2012). Dynamic sensor bias correction for attitude estimation using unscented Kalman filter in autonomous vehicle. Int. J. Innov. Comput. Inf. Control.

[40] Barrey E., Hermelin M., Vaudelin J., Poirel D., Valette J. (1994). Utilisation of an accelerometric device in equine gait analysis. Equine Vet. J..

[41] Auvinet B., Chaleil D., Barrey E. (1998). Accelerometric gait analysis for use in hospital outpatients. Revue du rhumatisme (English ed.).

[42] Brodie M., Walmsley A., Page W. (2008). Dynamic accuracy of inertial measurement units during simple pendulum motion: Technical Note. Comput. Methods Biomech. Biomed. Eng..

[43] Mariani B., Rochat S., Bula C., Aminian K. (2012). Heel and Toe Clearance Estimation for Gait Analysis Using Wireless Inertial Sensors. IEEE Trans. Biomed. Eng..

